# Bone density and genomic analysis unfold cold adaptation mechanisms of ancient inhabitants of Tierra del Fuego

**DOI:** 10.1038/s41598-021-02783-1

**Published:** 2021-12-02

**Authors:** Mikiko Watanabe, Renata Risi, Mary Anne Tafuri, Valentina Silvestri, Daniel D’Andrea, Domenico Raimondo, Sandra Rea, Fabio Di Vincenzo, Antonio Profico, Dario Tuccinardi, Rosa Sciuto, Sabrina Basciani, Stefania Mariani, Carla Lubrano, Saverio Cinti, Laura Ottini, Giorgio Manzi, Lucio Gnessi

**Affiliations:** 1 Section of Medical Pathophysiology, Food Science and Endocrinology, Department of Experimental Medicine, Sapienza University of Rome, Rome, Italy; 2 Department of Environmental Biology, Sapienza University of Rome, Rome, Italy; 3 Department of Molecular Medicine, Sapienza University of Rome, Rome, Italy; 4 MRC Centre for Neuropsychiatric Genetics and Genomics, Cardiff University, Cardiff, Wales UK; 5 Nuclear Medicine Unit, IRCCS Regina Elena National Cancer Institute, Rome, Italy; 6 Natural History Museum-University of Florence, Florence, Italy; 7 Italian Institute of Human Paleontology (IsIPU), Anagni-Rome, Italy; 8 Unit of Endocrinology and Diabetes, Campus Bio-Medico University of Rome, 00128 Rome, Italy; 9 Center of Obesity, Marche Polytechnic University, Ancona, Italy

**Keywords:** Evolution, Environmental sciences, Endocrinology

## Abstract

The Fuegians, ancient inhabitants of Tierra del Fuego, are an exemplary case of a cold-adapted population, since they were capable of living in extreme climatic conditions without any adequate clothing. However, the mechanisms of their extraordinary resistance to cold remain enigmatic. Brown adipose tissue (BAT) plays a crucial role in this kind of adaptation, besides having a protective role on the detrimental effect of low temperatures on bone structure. Skeletal remains of 12 adult Fuegians, collected in the second half of XIX century, were analyzed for bone mineral density and structure. We show that, despite the unfavorable climate, bone mineral density of Fuegians was close to that seen in modern humans living in temperate zones. Furthermore, we report significant differences between Fuegians and other cold-adapted populations in the frequency of the Homeobox protein Hox-C4 (HOXC4) rs190771160 variant, a gene involved in BAT differentiation, whose identified variant is predicted to upregulate HOXC4 expression. Greater BAT accumulation might therefore explain the Fuegians extreme cold-resistance and the protection against major cold-related damage. These results increase our understanding of how ecological challenges have been important drivers of human–environment interactions during Humankind history.

## Introduction

Subsequently to the dispersal of *Homo sapiens* out of Africa during the Paleolithic^1^, human populations required adaptations to diverse climatic conditions^2^ achieved through morphological and cultural adjustments and metabolic mutations^3^, such as brown adipose tissue (BAT) upregulation in cold climates^4^. BAT is a highly heterogeneous energy-expending tissue that generates heat, characterized by extraordinary plasticity^5^. BAT helps maintaining core temperature in a cold environment without shivering. In humans, BAT is found primarily in infants and young children. Defined regions of functionally active BAT are present in some but not all adults (3–10%)^6,7^ in thermoneutral conditions (approximately 18–24 °C) and may be quantified noninvasively with the use of (^18^)F-fluorodeoxyglucose ((^18^)F-FDG) positron-emission tomographic and computed tomographic (PET-CT) scans. BAT negative individuals do not have measurable metabolically active BAT, provided that the temperature conditions the BAT measurement was carried out are given. For humans in typical living conditions (with clothing and control over environmental temperature) the contribution of BAT to total energy expenditure is believed to be very small. BAT can be activated with various levels of intensity depending on sex, age, and ethnicity by cold exposure^6,8^.

The interest in understanding the mechanisms of cold adaptation comes both from obvious anthropological and evolutionary implications, and from theories that attribute to populations with higher BAT activity an increased resistance to obesity and diabetes^9^. When Europeans reached Tierra del Fuego in 1520, it was inhabited by four ethnic groups: the Yamana/Yaghan, Haush and Kaweskar/Alakaluf, all hunter-fisher-gatherers spending a significant portion of their time in the canoes and swimming, and the Selknam, terrestrial hunters, altogether generally grouped under the term Fuegians^10^. These ethnic groups were identified as different communities in the XX century^11^. The descendants of Fuegians show a population continuity as supported by the genetic affinity of modern Yamana and Alakaluf with ancient individuals from their respective regions and a mixture of European ancestry reflecting post-colonial admixture^12–14^. What is known of the ancient inhabitants of Tierra del Fuego mostly comes from navigators and ethnographers’ reports^15,16^. All were nomadic hunter-gatherers, and they had an exceptional cold resistance: despite the extremely harsh climate, they did not use any closed clothing, and open fires and animal fat smeared on bare skin appeared inadequate means of protection to those describing them^10^.

The climate in this region is inhospitable. It is a subpolar oceanic climate (Köppen climate classification Cfc) with short, cool summers and long, wet, moderate winters: the northeast is characterized by strong winds and little precipitation, in the south and west it is very windy, foggy, and wet for most of the year and there are very few days without rain, slush, hail or snow. Temperatures are steady throughout the year and across several centuries^17^: in Ushuaia they hardly surpass 9 °C (50 °F) in summers and average 0 °C (30 °F) in winters. Snowfall can occur in summer. The cold and wet summers help preserve the ancient glaciers.

Cultural and physiological mechanisms implicated in cold adaptation include clothing and shelter, and bodily changes such as peripheral vasoconstriction, muscle and subcutaneous fat-mediated insulation, lower surface area-to-mass ratio^18^, thyroid hormone levels variation^19,20^, high-fat and -protein diet^19,21^, BAT over-activity, and white adipose tissue browning^18,22,23^. In humans, BAT increases upon cold exposure^6,24^, and it generates heat via upregulation of uncoupling protein-1 (UCP1), increasing basal metabolic rate (BMR)^25^. Interestingly, in 1960 Hammel studied nine Fuegians belonging to the Alakaluf group, showing that their BMR was about 160% of that reported for the cold exposed "average white man" (p. 24)^26^. This report raised the suspicion that the Fuegians might have had increased BAT activity at the root of their amazing cold adaptation.

Beyond the interest in the role of BAT in thermoregulation and as a target for the prevention and treatment of obesity and type 2 diabetes, cold-activated BAT is also positively related to bone mineral density (BMD)^27^ and femoral cross-sectional area (CSA)^28^. BAT volume parallels BMD in some^29^, although not all reports^30^, and predicts femoral CSA and cortical bone area (CBA)^28,31^. Moreover, BAT-impaired cold-exposed mice show more bone loss than WT via β2 adrenergic receptor activation, this loss being reduced through β2 adrenergic receptor pharmacologic blockade^32^; and UCP1 knock-out (KO) mice under permanent cold stress have lower bone formation and mineralization compared to WT^33^. These altered bone phenotypes are not seen in thermoneutral UCP-1 KO and WT mice, indicating that BAT may prevent, although not eliminate, an otherwise massive cold-induced bone loss^33^. Finally, it is well established that populations living in cold geographic areas have reduced bone density despite milder temperatures during summers^34–36^, and recent evidence shows that they also have increased BAT activity^37^.

To test the hypothesis that BAT could have contributed to the exceptional cold adaptation of the ancient Fuegians, and in the absence of more direct means to investigate BAT in this population, we leveraged the reported correlation between bone and BAT activation by evaluating bone morphology and density of the skeletal remains of the collection of Fuegians preserved in the Museum of Anthropology ‘G. Sergi’ of the Sapienza University of Rome (Italy). To deepen the possible link, we then analyzed genomes of this population available in public repositories for variants in genes involved in BAT modulation.

## Results and discussion

To confirm BAT and bone physiology interconnection, widely reported in animal models but less established in human beings, we first explored the correlation between BAT and femoral cortical thickness in a living population composed of 34 BAT expressing individuals from a previously described large cohort^6^. In line with previous reports, BAT volume and activity were directly related to femoral CBA (Table S1). Moreover, BAT volume independently predicted CBA after height and muscle area (Table S2). After confirming this association in the living population, we analyzed Fuegians’ bones for comparison.

**Figure. 1:**
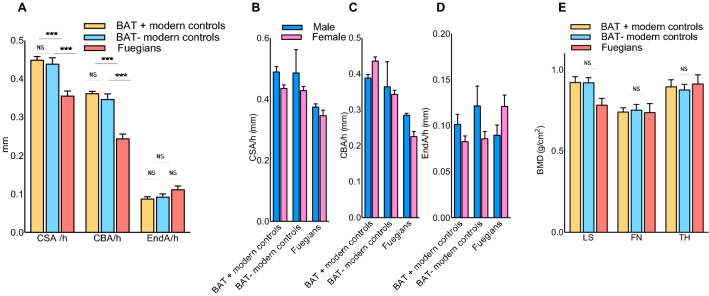
Bone geometry and density comparison of a modern BAT+ and BAT− population living in a temperate area and cold adapted Fuegian skeletal remains. (**A**) Living subjects expressing Brown Adipose Tissue (BAT+) are not significantly different in terms of femoral geometry [height (h) standardized Cross sectional (CSA), Cortical Bone (CBA) and Endocortical Area (EndA)] compared to living subjects not expressing BAT (BAT−). Conversely, Fuegians CSA and CBA are lower, with EndA not being significantly different. (**B**–**D**) Height standardized CSA, CBA, and ENDa stratified by gender. (**E**) Living subjects expressing Brown Adipose Tissue (BAT+) are not significantly different in terms of bone mineral density (BMD) compared to living subjects not expressing BAT (BAT−) and the Fuegians at both lumbar and femoral level. LS, Lumbar spine; FN, Femoral Neck; TH, Total Hip; ***P < 0.001; ns, not significant.

The Fuegians skeleton series preserved in Rome is composed of 14 complete skeletons; it represents one of the largest collections outside Argentina and is in an exceptionally good state of preservation. Twelve adults were included, and their bones were analyzed (Table S3). Fuegians femoral CBA and CSA were significantly lower than those of BAT positive and negative living humans, whereas the endocortical bone area (EndA) was comparable (Fig. 1A). Gender stratification suggests similar differences across groups, although the small sample size does not allow for analysis (Fig. 1B–D). A dual-energy X-ray-absorptiometry (DXA) of the Fuegians femur and lumbar spine remains showed no different BMD compared with a subpopulation of 12 matched BAT expressing and 12 non-expressing subjects living in temperate zones (mean yearly temperature of 16 °C) and selected among the cohort described above^6^ (Fig. 1E).

The observation that, despite their exposure to environmental conditions unusually detrimental for bone^34–36^, the Fuegians showed similar BMD compared to modern subjects living in temperate zones supports the hypothesis that these peoples had some sort of mechanism capable of protecting their bones from even more negative consequences. Similar to what observed in mouse models, where BAT is capable of partially limiting bone loss consequent to chronic cold exposure^38^, the hypothesis that a high expression of BAT could have contributed to protect the bones of the Fuegians in terms of BMD, though not enough to avoid thinner cortical bone, was still standing, and led us to test whether the Fuegians might have had an abundance of BAT through a genomic approach.

We therefore analyzed available genomes of this population, and we obtained reliable results for five of the 11 ancient Fuegian genomes retrieved, of which three were Yamanas, one was Selknam and one Alakaluf^39^. First, we selected a panel of genes involved in the differentiation, metabolism, and accumulation of BAT, as these pathways are reasonably the ones responsible for differential thermoregulation and thermogenesis in human populations^22^. The panel was identified by Sazzini et al. based on literature revision and after exploration of protein–protein interactions in the String database (http://string-db.org/). Some genes with unknown biological functions were included if they had been associated with obesity in genome-wide association studies^22^. Coding and non-coding variants in these genes involved in BAT functional pathways were identified in the Fuegian genomes (Dataset S1). All variants found in the Fuegians were searched in a control population of 14 individuals including 10 Siberians, 2 Athabascan and 2 Greenlanders^39^. These individuals, all living in cold areas of the globe (modern Alaska, Greenland and Siberia), were selected for the less striking cold adaptation leading to different lifestyle and habits, such as the use of warm clothing, compared to Fuegians, whose main protections from the harsh climate were open fires, animal fat smeared on bare skin and at most a small piece of fur over the shoulders.

Interestingly, a coding variant previously associated with cold adaptation^22^, the cAMP-dependent protein kinase type II-beta regulatory subunit (*PRKAR2B)* rs75385144, was identified in four out of five Fuegians, with an allelic frequency of 0.4 (Table 1). This candidate variant is reported with a frequency of 0.33 in the gnomAD database, however it has a very low frequency in the African population (0.07) compared with European, American and Asian populations (0.3–0.4)^22^. Notably, the variant was found in a frequency comparable to Fuegians in our control population including Siberians, Athabascan and Greenlanders (11 out of 14 individuals, allelic frequency of 0.39, p-value for comparison = 0.95). These results suggest that this variant might be implicated in cold adaptation, as the low frequency in African population suggested, but not in different ways across populations.

**Table 1:** Summary of relevant genomic variants.

rs number	Gene	Nucleotide change	Consequence	Number of variant carriers among Fuegians	Number of variant carriers among controls	gnomAD total allele frequency	In silico prediction	Reference
rs75385144	*PRKAR2B*	NM_002736.3:c.96G>T	Synonymous variant	4/5	11/14	0.33	Polymorphism	^12^
rs2493270	*PRDM16*	NG_029576.1:g.369684A>G	3 Prime UTR variant	3/5	0/14	0.07	Polymorphism	**–**
rs190771160	HOXC4	NG_029818.1:g.26103T>C	Intron variant	4/5	1/14	0.003	Likely effect on regulatory regions	**–**

PRKAR2B, Protein Kinase CAMP-Dependent Type II Regulatory Subunit Beta; PRDM16, PR domain containing 16; HOXC4, Homeobox protein Hox-C4; UTR, untranslated region.

**Figure 2:**
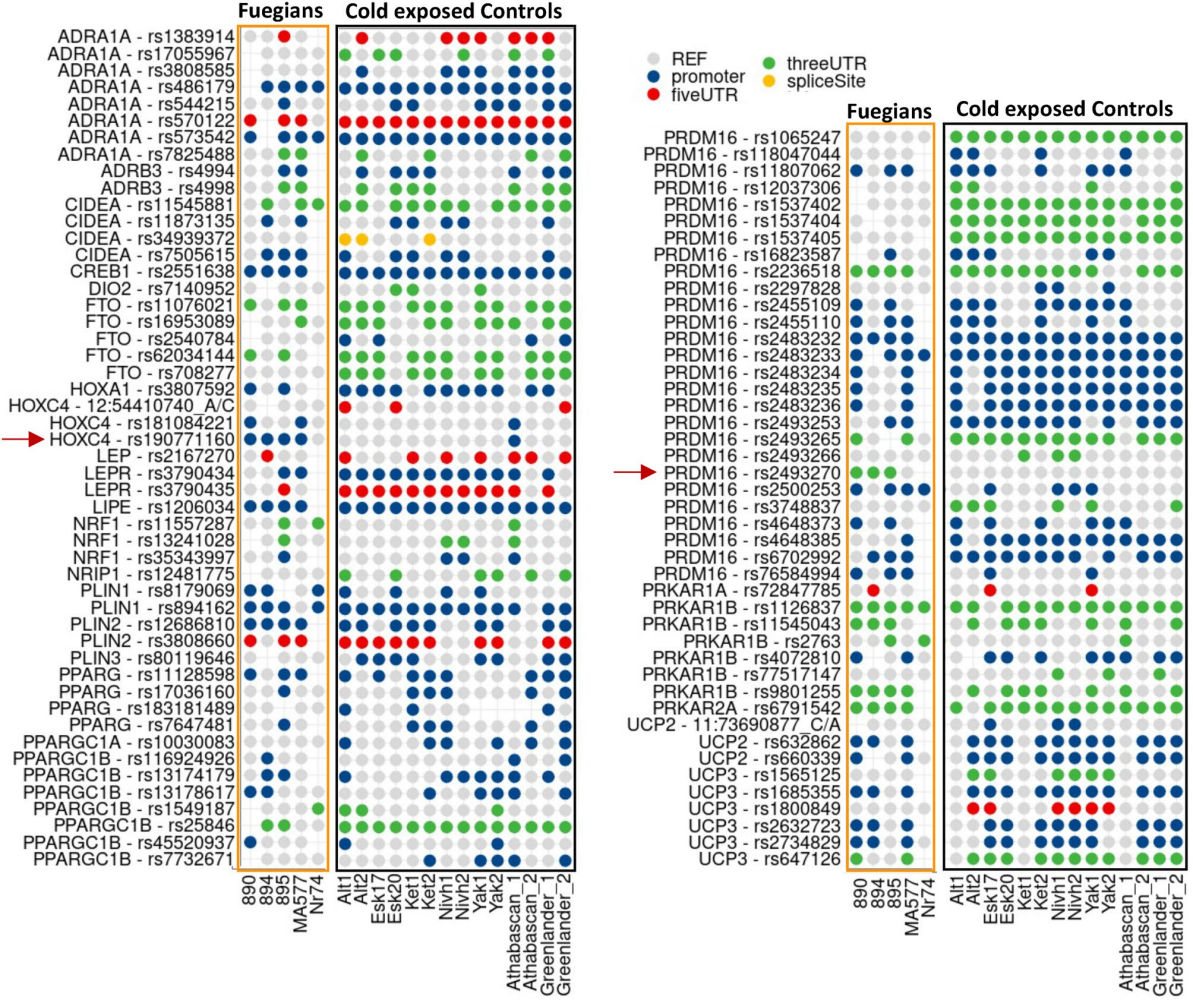
Non-coding variants of a panel of 28 genes involved in BAT functional pathways identified in at least 2 Fuegian genomes. All variants found in the Fuegians (890, 894, 895, MA577, Nr74) were searched in control population of 14 individuals including 10 Siberians (Alt1, Alt2, Esk17, Esk20, Ket1, Ket 2, Yak1, Yak2), 2 Athabascan (Athabascan_1, Athabascan_2) and 2 Greenlanders (Greenlander_1, Greenlander_2). Red arrows: variants of interest described within the manuscript.

Moreover, a statistically significant difference in frequency between Fuegians and the control population emerged for two non-coding variants, PR domain containing 16 (PRDM16) rs2493270 and Homeobox protein Hox-C4 (HOXC4) rs190771160, both genes being involved in BAT differentiation^22,40^ (Table 1, Fig. 2).

PRDM16 is present in brown adipocytes and is able to upregulate UCP-1 expression, thus increasing the activity of BAT. Its overexpression is also associated with browning in mouse models^41^. Its variant rs2493270 was found in three out of five Fuegians and was not identified in controls (p = 0.002) (Table 1, Fig. 2). In gnomAD, the variant is reported with a very low frequency in the Finnish population, and in slightly higher frequencies in Latinos, East Asians and Africans. The variant is an A to G substitution within the 3′ UTR region of the PRDM16 gene, predicted as likely benign by in silico tools, although a role in modifying genomic interactions and miRNA binding sites cannot be excluded with possible functional consequences on beige and brown adipocytes.

HOXC4 rs190771160 was found in four out of five Fuegians and only one out of 14 controls (p = 0.0015) (Table 1, Fig. 2). This variant is reported with a very low frequency in all modern populations whose genomes are deposited in gnomAD database. The variant is a C to T substitution within a highly phylogenetic conserved intronic/promoter region of HOXC4. Intriguingly, all of the interrogated in silico tools consistently predicted a very likely causative effect in affecting histone modifications, mainly tri-methylation at lysine 27 of histone H3 (H3K27me3). Moreover, the transcription factor USF1 binding site seems to be affected by the variant as predicted by the same in silico tools (Table 1). Enhanced expression of HOXC4 has been linked to the accumulation of fat droplets within brown adipocytes^40^. Anticipated to affect H3K27me3 and USF1 binding, both inhibiting transcription^42,43^, the presence of rs190771160 is likely to upregulate HOXC4 expression, leading to enhanced BAT proliferation in those carrying the variant.

We are aware that our approach suffers of some limitations. First, the studies on human subjects were conducted retrospectively: the availability of DXA scans performed at a similar time compared to the PET scan recorded for the study subjects was minimal, making our contemporary human sample small. Moreover, some parameters, such as smoking and alcohol habitual consumption, possibly affecting bone health were not available. Contrary to the association between bone geometry and BAT activity, which we were able to confirm in our BAT expressing living population (supplementary tables), we were unable to extract similar results regarding BMD due to the small number of BAT expressing living subjects with a DXA scan available. Published preliminary data suggest that the well-established link between BAT and BMD in rodent models might be true in human subjects as well, reason why we speculated that the finding that the cold exposed Fuegians BMD was similar to that of those living in temperate areas could be at least partially attributable to increased BAT activity. Clinical studies focusing on the link between bone density and BAT, with an adequate sample size, are warranted to fill the gap in literature. Moreover, in our sample, gender stratification showed a possible difference between male and female, which was not explored further due to the small population. Additionally, age and physical demand may as well have played roles in determining the Fuegians bone characteristics^44–46^. Moreover, it is hard to determine to which extent the Fuegians resistance to cold lied in the bodily changes as opposed to sufficient cold protection behaviours (shelter, fires, rudimental clothing), the latter more commonly a research issue in scientific literature. As previously mentioned, the habits of the ancient inhabitants of Tierra del Fuego were described by navigators and ethnographers, and the reports are charming but sometimes incomplete or contradictory. Again, further studies are warranted to investigate on these aspects. Second, for obvious reasons, there is small availability of Fuegian skeletons, and indeed the Roman collection is among the richest worldwide, together with collections housed in museums and research institutions of Argentina and Chile, making our sample generally small but actually wider than usual. Third, diagenesis, possibly hindering the bone analysis, cannot be ruled out. However, previously conducted analysis on the skeletal remains included in the present study showed and exceptional state of preservation as reflected by the collagen percentage and the stable carbon and nitrogen isotope ratios reported^17^. Fourth, the identified control populations for genomic comparison are not ideal, as they were living at a mean winter temperature approximately 10 °C lower than that of the Fuegians. However, the latter had little clothing to protect themselves as opposed to the inhabitants of subpolar northern regions, making them in our opinion the most suitable controls among other contemporary populations. Certainly, we cannot rule out that some population, better suited for such comparison, might be available, and further studies comparing the Fuegians with them would be ideal.

Overall, both the genomic data and bone analysis of the Fuegians suggest that this population might have been characterized by large BAT depots. However, the possibility that the Fuegians could have gained their resistance to cold through other functional pathways cannot be definitely ruled out. Likewise, we cannot exclude that factor such as muscle mass, whose positive correlation with bone mass is established, may have contributed to their better-than-expected BMDs. However, even if this was the case, our hypothesis could be maintained since the myokines derived from skeletal muscle after contraction or cold exposure (i.e. irisin) seem to have profound effects on enhancing bone mass and reinforcing the BAT phenotype^47–50^.

In conclusion, although based on a small sample, our findings may confirm the hypothesis that the extraordinary cold-adaptation of the Fuegians, already noted by the nineteenth-century explorers, was due to an exceptional BAT accumulation. Our results can be framed as an example of biological adaptation as opposed to the cultural and technological aids that allowed populations to occupy new environments without having to first evolve biological adaptations to them. Whether this distinctive adaptive mechanism of the Fuegians to cold played some role in the human macroevolution deserves further studies.

## Materials and methods

### Study populations

#### Fuegian remains

The skeletal remains of 12 adult Yàmanas (4 male, 8 female) from Tierra del Fuego were selected from the collection of the Museum of Anthropology "G. Sergi" of the Sapienza University of Rome (Italy) (Supplementary Table 3), which includes fifteen complete or almost complete skeletons (13 adults, 1 juvenile, 1 infant) in good state of preservation as reflected by previous analysis^17^.The selection criteria included the availability of femur and lumbar vertebras and estimated age of 25 years old or older as proof of achievement of adult bone structure. The collection was recovered by the Italian explorer captain Giacomo Bove. Bove recovered two skeletal collections of Fuegians in 1881 and 1883 stored respectively in the Museo di Storia Naturale in Florence and the Museo di Antropologia G. Sergi of Rome. Captain Bove recovered thirteen of the skeletons preserved in Rome during his second trip, while the further samples were later acquired by the Rome Museum through a donation. The history of the recovery and the composition of the assemblages are fully described in a paper by Marangoni et al.^51^. Both assemblages are described in Bove’s reports^15^, and were given to the captain by Yámana settled in the area of Yendegaia, near Ushuaia where in the 1860’s an Anglican Mission was established^17^. Considering Bove’s notes, those human remains refer to family members to whom the natives related suggesting a tentative attribution to the first half of the nineteenth century^17^. This fact suggests that the skeletons in the roman collection might belong to Fuegians who lived at a time when contacts with European and North American sealers became relatively more systematic although a steady systematic contact with Europeans would have taken place from 1843 onwards when the Chilean government sent an expedition whose aim was to establish a new city that would serve as a centre for the economic development of the region. Thus, we do not know whether, according with previous observations^12–14^, the remains of the roman collection belong to individuals modern enough to show a mixture of European ancestry (reflecting post-colonial admixture).

#### Living population

We retrospectively selected a population of 217 patients expressing F-FDG BAT among a total of 6454 patients who underwent 8004 consecutive 18F-FDG PET/CT scans from January 2007 to June 2010 at the Istituto Nazionale dei Tumori Regina Elena (Rome, Italy; 41.81°N 12.45°W). The modality of BAT expression detection is described elsewhere^6^. Briefly, patients were considered to have BAT when the following criteria were met: (1) ^18^F-FDG uptake was in the paravertebral/cervical, supraclavicular, mediastinal, paravertebral/dorsal, and abdominal or perirenal areas, unrelated to muscle, joints and pathological findings; (2) ^18^F-FDG uptake had a maximum standardized uptake value (SUV) of 2.0 g/ml or greater (an indicator of ^18^F-FDG uptake intensity); and (3) the tissue corresponded to the density of adipose tissue on CT (– 250 to – 50 Hounsfield units)^6^. Among the 217 patients with BAT depots, individuals younger than 25 years old or who underwent PET/CT scans for malignant diseases and received the last treatment for malignant diseases within 1 year before the scan were excluded, as where subjects whose diagnosis and/or treatment timings were unknown. We also excluded those with diabetes, renal failure, and under steroids or osteoporosis treatment at the time of PET/CT. Moreover, a gender, age and BMI matched control population of BAT negative subjects was selected among the same population following the same exclusion criteria stated above. To control for BMI difference, given the reported lean body mass of the Fuegians, we only selected patients with a BMI ≤ 25 kg/m^2^.

Among the patients enrolled in the present study, a subpopulation was subsequently selected to be age, BMI and gender matched to those estimated for the fuegian skeletal remains adopting the following criteria: available Dual energy X-ray absorptiometry (DXA) scan performed within 1 year of the PET scan with a Hologic scanner.

### Positron emission tomography/computed tomography (PET/CT) of living humans

PET/CT scans were performed using a Biograph 16 High Rez PET/CT scanner (Siemens AG, Munich, Germany). Patients came from the temperate metropolitan area of Rome and were instructed to fast overnight for at least 12 h before the scan and to abstain from carbohydrates and very fatty foods consumption, nicotine, caffeine, or alcohol intake for the preceding 24 h. Room temperature water was allowed at all times. Since their arrival to the hospital, the patients were in an air-conditioned environment at about 22 °C. After the injection of ^18^F- fluorodeoxyglucose (FDG) the patients rested at 24 °C for 1 h before undergoing the PET/CT scan. Data on gender, BMI, age, plasma glucose level and malignancy status (active: PET/CT scan positive for malignancy; not active = PET/CT scan negative for malignancy) were obtained for all patients. Parameters measured from PET/CT scans included, in addition to BAT characteristics: cross-sectional area (CSA, mm2), cortical bone area (CBA, mm2), endocortical bone area (EndA, mm2), muscle area (MA, mm2) taken at proximal-shaft diaphysis, 1 cm distal to the lesser trochanter. These were calculated with the use of ImageJ 1.52i, Wayne Rasband, National Institutes of Health, USA.

### Computed tomography (CT) of Fuegians femurs

For each Fuegian skeleton, the right femur was analyzed using a commercial CT scanner (Revolution, GE Healthcare). Before the scan, femurs were oriented in standardized anatomical planes. Diaphyseal structural properties were calculated from DICOM files with the use of ImageJ 1.52i (Wayne Rasband, National Institutes of Health, USA). Parameters measured included: cross-sectional area (CSA, mm2), cortical bone area (CBA, mm2), and endocortical bone area (EndA, mm2) taken at proximal-shaft diaphysis, 1 cm distal to the lesser trochanter. In order to control by body size, CSA, CBA and EndA were standardized by estimated height^52^. Briefly, height was calculated through Manouvrier tables using the femur, humerus, ulna and radius of the Fuegians, the mean of all estimations was taken as estimated height for each Fuegian skeleton. Then, CSA, CBA and EndA were divided by estimated height in the case of the Fuegian skeletons, and actual height in the case of the living population.

### DXA of femur and lumbar spine of Fuegians

Lumbar spine (LS, L1–L4), femoral neck (FN) and total hip (TH) BMD of Fuegians femur and lumbar vertebrae remains were measured through DXA (QDR Discovery Acclaim, Hologic Inc., Waltham, MA, USA). Before DXA scanning, femurs of Fuegians were oriented in standardized anatomical planes and the lumbar vertebrae placed in a rack that allowed them to be aligned, using rice as a soft tissue proxy^53^.

### Genome analysis

Alignment data from Raghavan et al.^39^ were downloaded from http://www.cbs.dtu.dk/suppl/NativeAmerican/. Data corresponding to 8 Fuegians and 14 distinct Native Americans individuals exposed to cold temperatures were identified and extracted. Read statistics per sample and alignment methods are shown in the Supplementary Materials for Raghavan et al.^39^.

Since the individual Fuegians libraries have a variable endogenous content (from 0.6 to 23.8% of the total number of reads mapped to the human genome) and a low sequencing depth (average depth from 0.003 to 1.7)^39^, samples with less than 5 million of mapper reads were removed prior to further analyses. The remaining 5 fuegian individuals included 3 Yamana (~ 47 M of average mapped reads and ~ 21% of endogenous content), 1 Selknam (81.5 M of mapped reads and ~ 16% of endogenous content) and 1 Alakaluf (~ 15 M of mapped reads and ~ 1.4% of endogenous content).Genotypes were called both per-sample and in a multi-sample approach by using GATK (v.4.1.2.0)^54^ and modelled after the GATK Best Practices Workflows and the parameters used in Raghavan et al.^39^. Briefly, variants were called per-sample using HaplotypeCaller in GVCF mode and prepared for filtering (tools involved: CombineGVCFs, GenotypeGVCFs). Variants were extracted, filtered and ricalibrated for QualByDepth, RMSMappingQuality, MappingQualityRankSumTest, ReadPosRankSum, FisherStrand, ReadPosRankSumTest and HaplotypeScore using the HapMap 3.3, and dbSNP138 resources with priors 15 and 2, respectively.

The filtered variants were imported and annotated using R packages vcfR (v.1.12.0), GenomicFeatures (v.1.42.1) and VariantAnnotation (v.1.36.0) and the gencode annotation v.19 as reference. SNPs located in coding, promoter and 5′/3′ UTR regions of a panel of 28 genes directly involved in BAT metabolism from Sazzini et al.^22^, were selected and further investigated (S1).

Candidate variants were further characterized based on possible functional effect as predicted by in silico analysis using MutationTaster^55^and RegulationSpotter^56^.These are online applications performing several in silico tests on both DNA and protein level ultimately estimating the impact of the identified variant, such as functional consequences of synonymous or intronic mutations up to amino acid substitutions, deletion and insertion of sequences, or variants within the intron–exon border.

### Ethics statement

Data relative to the study cohort of living humans was collected from a database previously used to report BAT prevalence in central Italy^6^. The project had been approved by the Institutional Review Board of the Dipartimento di Prevenzione e Diagnostica Oncologica, UOC di Medicina Nucleare, Istituto Nazionale Tumori Regina Elena, Roma. The data were analyzed anonymously. Written informed consent was given by the patients or from the next of kin or caretakers for their information to be stored in the hospital database and used for research. All methods, including human remains handling and storage, were carried out in accordance with relevant guidelines and regulations. All efforts were made to minimize damage to human remains, and data will be made available upon reasonable request to allow critical re-examination of scientific findings^57^.

### Statistics

Statistical analysis was performed using SPSS 25.0 (SPSS, Inc., Chicago, IL). Data are expressed as mean ± standard deviation for normally distributed variables. Variables were tested for normality of distribution using Shapiro–Wilk test. Variables that were not normally distributed were log-transformed. Comparisons between groups were performed using the paired Student's *t*-test or Wilcoxon test as appropriate. Relationships between variables were measured by Pearson’s correlation coefficient. Stepwise regression modeling was used to determine predictors of CBA. A chi-square test was performed to compare variant frequencies between Fuegians, Siberians/North Americans, and other modern populations from gnomAD database (https://gnomad.broadinstitute.org/). An α error of 0.05 was considered the threshold for statistical significance.

## Supplementary Information

Supplementary Information 1.

Supplementary Information 2.
