# Supplementary material for: Bone density and genomic analysis unfold cold adaptation mechanisms of ancient inhabitants of Tierra del Fuego
Source: Sci Rep. 2021 Dec 2;11:23290. doi: 10.1038/s41598-021-02783-1 (PMC8639971; doi:10.1038/s41598-021-02783-1)
Supplement: Supplementary file 1 — Supplementary Information 1. [file 41598_2021_2783_MOESM1_ESM.pdf]

Supplementary Information for

Bone density and genomic analysis unfold cold adaptation mechanisms of ancient inhabitants of Tierra del Fuego

Mikiko Watanabe[1§*], Renata Risi[1§], Mary Anne Tafuri[2], Valentina Silvestri[3], Daniel D'Andrea[4], Domenico Raimondo[3], Sandra Rea[5], Fabio Di Vincenzo[6], Antonio Profico[2], Dario Tuccinardi[7], Rosa Sciuto[5], Sabrina Basciani[1], Stefania Mariani[1], Carla Lubrano[1], Saverio Cinti[8], Laura Ottini[3], Giorgio Manzi[2#] and Lucio Gnessi[1*#]

[1] Department of Experimental Medicine, Section of Medical Pathophysiology, Food Science and Endocrinology, Sapienza University of Rome, Rome, Italy.
[2] Department of Environmental Biology, Sapienza University of Rome, Rome, Italy.
[3] Department of Molecular Medicine, Sapienza University of Rome, Rome, Italy
[4] MRC Centre for Neuropsychiatric Genetics and Genomics, Cardiff University, Cardiff, Wales, UK
[5] Nuclear Medicine Unit, IRCCS Regina Elena National Cancer Institute, Rome, Italy
[6] Natural History Museum - University of Florence, Italian Institute of Human Paleontology (IsIPU)
[7] Unit of Endocrinology and Diabetes, Campus Bio-Medico University of Rome, 00128 Rome, Italy
[8] Center of Obesity, Marche Polytechnic University , Ancona, Italy

[§] These authors contributed equally
[#] These authors contributed equally
*Corresponding author

Correspondence to:

Lucio Gnessi MD PhD, Department of Experimental Medicine, Section of Medical Pathophysiology, Food Science and Endocrinology, Sapienza University of Rome, Rome, Italy. Tel. +39 06 499 70 721
Mail: lucio.gnessi@uniroma1.it

This PDF file includes:

Tables S1 to S3
Legends for Datasets S1

Other supplementary materials for this manuscript include the following:

Datasets S1

Table S1. Adjusted Pearson correlations between bone geometry and BAT parameters.

**A**					
Height-adjusted	**CBA**	**CSA**	**MA**	**Bat Volume**	**Bat Activity**
**CBA**	1	0.896***	.558**	.392*	.417*
**CSA**		1	.359*	.294^	.290
**MA**		.	1	.173	.230
**Bat volume**				1	.903***
**Bat activity**					1
**B**					
Height & MA- adjusted	**CBA**	**CSA**		**Bat Volume**	**Bat Activity**
**CBA**	1	0.898***		.362*	.357*
**CSA**		1		.252	.228
**Bat volume**				1	.900***
**Bat activity**					1

A positive unadjusted correlation trend of CBA with BAT volume (p 0.07, r 0.307) and activity (p 0.07, r 0.315) was found. Adjusting the analysis for anthropometric parameters likely affecting femoral measures the following results emerged: **(A)** Height-adjusted partial correlation between CBA and BAT volume and activity in a BAT expressing living population (n=34) became significant, and a positive trend between CSA and BAT volume emerged. (**B)** Further adjusting by both height and muscle area (MA), the positive correlation trend between CSA and BAT indexes vanished, whereas the one between CBA and BAT volume and activity was maintained, suggesting that muscle plays a central role in bone remodeling. CSA, Cross-Sectional Area; CBA, Cortical Bone Area; MA, Muscle Area. ***P<0.001; **P<0.01; * P<0.05 level; ^ P<0.1

Table S2. Multiple regression analysis to assess predictors of cortical thickness.

A					
**Model**		**Standardized β**	**SE**	**Sig.**	**R**
**1**	MA	.723	.004	.000	.723
**2**	MA	.514	.005	.001	.781
	Height	.363	1.260	.013	
**3**	MA	.468	.005	.001	.812
	Height	.386	1.202	.006	
	BAT Volume	.224	.207	.047	
**4**	MA	.412	0.005	.002	.848
	Height	.516	1.211	.001	
	BAT Volume	.276	.195	.011	
	Age	.271	.619	.019	

Dependent Variable: CBA
Independent variables evaluated: MA, height, Bat Volume, Age

B				
	**Standardized β**	**SE**	**Sig.**	**R**
MA	.860	.005	.000	.731

Dependent Variable: CBA
Independent variables evaluated: MA, height, Age

(A) A stepwise multiple regression in a BAT expressing living population (n=34) including age, weight, height, MA, BAT volume as independent variables showed that BAT volume independently predicted CBA after height and MA, while (B) a stepwise multiple regression in a BAT negative living population (n=34) including the same independent variables showed that MA independently predicted CBA. These findings suggest that, in the absence of BAT, muscle mass closely influences bone morphology, while, when BAT is present, this acquires the strength of independent predictor of bone state. MA, Muscle Area; CBA, Cortical Bone Area.

Supplementary Table 3. Anthropometric characteristics, bone mineral density and geometry parameters of selected Fuegians bone remains

Sample ID	Sex	Estimated age at death	CSA mm²	CBA mm²	EndA mm²	LS BMD (g/cm²)	TH BMD (g/cm²)	FN BMD (g/cm²)
Fuegian 1	F	40+	583.68	312.16	271.53	Missing	0.666	0.533
Fuegian 2	F	20-30	472.86	313.69	159.17	0.709	0.893	0.714
Fuegian 3	F	40+	426.91	279.37	147.54	0.776	0.83	0.548
Fuegian 4	F	40+	492.03	259.56	232.48	0.642	0.893	0.721
Fuegian 5	M	40+	645.62	467.64	177.99	0.815	1.024	0.9
Fuegian 6	M	40+	562.22	442.24	119.98	0.788	0.986	0.808
Fuegian 7	F	40+	684.39	484.1	200.3	0.569	0.647	0.478
Fuegian 8	M	30-40	509.01	416.9	92.111	1.022	1.131	0.924
Fuegian 9	F	40+	411.8	321.7	90.104	0.817	1.076	0.873
Fuegian 10	F	20-30	505.57	348.71	156.86	0.877	1.043	0.858
Fuegian 11	F	40+	491.23	295.04	196.2	0.623	0.643	0.490
Fuegian 13a	M	40+	564.13	405.68	158.45	0.955	1.088	0.954

CSA, Cross-Sectional Area; CBA, Cortical Bone Area; EndA, Endocortical Area; LS, LumbarSpine; BMD, Bone Mineral Density; TH, Total Hip; FN, Femoral Neck; F, Female; M, Male.

Dataset S1 (separate file). Coding and non coding variants identified in the Fuegian population.
